# Quantitative assessment of placental alpha macroglobulin‐1 for predicting impending preterm delivery in asymptomatic women with a short cervix

**DOI:** 10.1111/jog.70071

**Published:** 2025-09-05

**Authors:** Yuki Nozaki, Kenji Imai, Rika Miki, Sho Tano, Kazuya Fuma, Seiko Matsuo, Takafumi Ushida, Hiroaki Kajiyama, Tomomi Kotani

**Affiliations:** ^1^ Department of Obstetrics and Gynecology Nagoya University Graduate School of Medicine Nagoya Japan; ^2^ Department of Obstetrics and Gynecology Japanese Red Cross Aichi Medical Center Nagoya Daini Hospital Nagoya Japan; ^3^ Maternal Fetal Health Laboratory, Research Institute Nozaki Tokushukai Hospital Daito Osaka Japan; ^4^ Laboratory of Bell Research Center‐Department of Obstetrics and Gynecology Collaborative Research Nagoya University Graduate School of Medicine Nagoya Japan; ^5^ Department of Obstetrics and Gynecology Hamamatsu University School of Medicine Hamamatsu Japan

**Keywords:** birth, obstetrics, premature, preterm

## Abstract

**Aims:**

Preterm delivery (PTD) is a leading cause of neonatal morbidity and mortality. Accurate prediction is crucial for optimizing clinical outcomes, particularly in women with a short cervix. Although fetal fibronectin (FFN) is widely used to predict PTD, placental alpha‐microglobulin‐1 (PAMG‐1) has gained attention for its potential to improve predictive accuracy. This study aimed to evaluate the utility of quantitative PAMG‐1 assessment for predicting impending PTD in asymptomatic women with a short cervix (≤25 mm) between 24 and 34 weeks of gestation.

**Methods:**

This observational cohort study analyzed 212 cervicovaginal fluid samples from 77 patients (132 from 49 singleton and 80 from 28 twin pregnancies). PAMG‐1 and FFN levels were measured, and multivariate logistic regression was performed to evaluate their association with PTD risk.

**Results:**

In singleton pregnancies, positive PAMG‐1 was independently associated with impending PTD, with odds ratios of 7.84 (95% confidence interval [CI], 2.02–30.50; *p* = 0.003) and 7.34 (95% CI, 2.75–19.60; *p* < 0.001) for PTD within 1 and 2 weeks, respectively. Quantitative PAMG‐1 showed a dose‐dependent relationship, with PTD risks increasing from 4.3% (<1000 pg/mL) to 50.0% (≥3000 pg/mL) within 1 week and from 10.0% to 90.0% for PTD within 2 weeks. In twin pregnancies, both PAMG‐1 and FFN showed limited predictive utility.

**Conclusions:**

This study highlights the potential of PAMG‐1 quantification as a valuable tool for refining PTD risk stratification, particularly in singleton pregnancies. While further prospective multicenter validation is needed, these findings provide new clinical insights for improving PTD management.

## INTRODUCTION

Preterm delivery (PTD) is a leading cause of perinatal mortality and morbidity worldwide, significantly contributing to long‐term health complications, including neurodevelopmental impairments and chronic illnesses in surviving infants. Despite advances in obstetric care, the global PTD rate has remained largely unchanged over the past decade, highlighting the urgent need for more effective management strategies.^1^, ^2^

Accurate prediction of PTD is critical for optimizing perinatal outcomes. Timely identification of women at risk allows for the administration of appropriate interventions, such as antenatal corticosteroid therapy to enhance fetal lung maturity, magnesium sulfate for fetal neuroprotection, and in utero transfer to facilities equipped for preterm neonatal care. Conversely, accurate identification of women not at imminent risk of PTD reduces unnecessary interventions, minimizing healthcare costs, potential adverse effects, and patient burden.^3^, ^4^ Sonographic cervical length (CL) measurement is widely recognized as a reliable and cost‐effective method for predicting PTD in asymptomatic women.^5^ However, its predictive accuracy can be further improved by incorporating biochemical markers. Several biomarkers, including fetal fibronectin (FFN) and several cytokines in cervicovaginal fluid (CVF), have been evaluated for their utility in assessing PTD risk.^6^, ^7^

Placental alpha‐microglobulin‐1 (PAMG‐1) has emerged as a novel biomarker due to its unique biochemical properties. PAMG‐1 is found in high concentrations in amniotic fluid and significantly lower concentrations in cervicovaginal discharge.^8^ Qualitative diagnostic tools, such as the PartoSure® test, which use a threshold of 1000 pg/mL for PAMG‐1 at CVF, have been used for predicting PTD. A recent systematic review and meta‐analysis demonstrated that, for the prediction of spontaneous PTD in symptomatic women, the positive predictive value (PPV) of PAMG‐1 surpasses those of FFN and phosphorylated insulin‐like growth factor binding protein‐1.^6^

In contrast to many other countries, PAMG‐1 testing (e.g., AmniSure® or PartoSure®) is not approved or routinely used in Japanese clinical practice. Therefore, no prior data exist evaluating its utility in Japanese populations. Moreover, although quantitative tests for FFN have shown superior predictive accuracy compared to qualitative tests,^9^ the potential benefits of quantitative PAMG‐1 assessment remain unexplored. Quantitative measurement of PAMG‐1 may further enhance its predictive capacity, providing a valuable addition to PTD risk assessment tools.

Therefore, the present study aims to validate the predictive value of PAMG‐1 using the established 1000 pg/mL threshold in a Japanese cohort of asymptomatic women with a short cervix and to explore the additional utility of quantitative PAMG‐1 measurements for improving the prediction of preterm labor. By addressing existing knowledge gaps, we aim to provide insights that could improve the prediction of PTD in this population.

## MATERIALS AND METHODS

### Patient enrollment and clinical data

This retrospective observational cohort study was approved by the Institutional Review Board and Ethics Committee of Nagoya University Graduate School of Medicine (approval number 20220008). Written informed consent was obtained from all the enrolled patients.

At our institution, CL measurement is routinely performed in asymptomatic pregnant women around 24 weeks of gestation, and again between 28 and 30 weeks, as part of our standard prenatal assessment. For women admitted due to a short cervix, CL measurements are performed once weekly during hospitalization to monitor cervical changes. However, as this was a retrospective study, some variability in timing occurred based on the attending physician's discretion. In some cases, such as when patients reported anxiety or concern, additional CL assessments were performed more frequently.

Between May 2020 and March 2023, we collected CVF samples from pregnant women who were admitted to our hospital with a diagnosis of a short cervix (≤ 25 mm), typically identified between 28 and 32 weeks of gestation. Following admission, CVF sampling was performed repeatedly for ongoing surveillance until 34 weeks of gestation. All included women had no pregnancy complications, such as major congenital fetal anomalies, vaginal bleeding, bacterial vaginosis, rupture of the fetal membrane, or malignancy. In total, 280 CVF samples were collected from 116 patients (164 samples from 75 singleton pregnancies and 116 samples from 42 twin pregnancies). We excluded the samples collected from women with iatrogenic preterm birth. Consequently, 212 CVF samples from 77 patients (132 from 49 singleton pregnancies and 80 from 28 twin pregnancies) were included in the present study. All CVF samples were obtained from women who were asymptomatic at the time of collection. Although occasional Braxton Hicks contractions were observed in some cases, there were no painful, regular uterine contractions indicative of active labor, and no cases exhibited vaginal bleeding or clinical signs of membrane rupture. The other clinical data of the included patients were extracted from hospital records. In this study, infertility treatment was defined as artificial insemination or in vitro fertilization. In most cases, vaginal progesterone (200 mg) was administered intravaginally for PTD prevention at the discretion of the attending physician. Histological chorioamnionitis was defined as the infiltration of polymorphonuclear leukocytes into the chorioamniotic membrane.

### Placental alpha macroglobulin‐1 and fetal fibronectin in cervicovaginal fluid

As part of our clinical practice, we routinely conducted FFN tests in hospitalized pregnant women. Simultaneously, we collected CVF samples from those who met the aforementioned criteria. Before collection of the CVF, CL was measured transvaginally by trained obstetricians using standardized techniques recommended by the International Society of Ultrasound in Obstetrics and Gynecology (ISUOG) Practice Guidelines for preterm birth screening; either the two‐step method or the tracing method was employed to enhance accuracy and minimize inter‐observer variability.^10^ The tests, collection, and scanning were repeated as required for pregnancy surveillance. The CVF was obtained and stored using the previously described method.^7^ Briefly, sterile Dacron swabs were placed on the posterior fornix, subsequently deposited in 0.55 mL solvent solutions, and stored at −80°C until analysis. To determine the PAMG‐1 concentration, commercially available ELISA kits (SunLong Biotech Co., China) were used according to the manufacturer's protocol. To measure FFN level, we used a fibronectin collection kit (SRL Co., Ltd., Tokyo, Japan) according to the manufacturer's instructions, and the measurements were performed commercially by SRL. All measurements were duplicated, and the mean values were used in the analysis.

### Data analysis and statistics

Statistical analyses were performed using R version 4.2.1 software. Statistical significance was set at *p* < 0.05. Maternal and neonatal/delivery characteristics were compared among each group using Fisher's exact test for categorical variables and Mann–Whitney *U* test for continuous variables. Multivariate logistic regression analyses were performed to assess potential relationships of various characteristics including the positive FFN (≥50 ng/mL) and PAMG‐1 (≥1000 pg/mL) to PTD within 1 to 2 weeks after the CVF sampling (named “Impending PTD”). Each adjusted odds ratio (OR) and 95% confidence intervals (CIs) were evaluated after adjusting for maternal age at delivery (≥35 years), parity, smoking, history of PTD, and CL (≥15 mm). To stratify the risk of PTD within 1 to 2 weeks after CVF sampling, thresholds of 1000, 2000, and 3000 pg/mL for the PAMG‐1 level were set, and the FFN level was divided into groups of <50, 50–149, and ≥150 ng/mL. We considered these to be pragmatic and memorable thresholds. The correlation between FFN and PAMG‐1 levels was also examined. Receiver operating characteristic (ROC) curves were generated to compare the abilities of PAMG‐1, FFN, and their combination. In addition, the Cochran‐Armitage trend test was used to evaluate dose‐dependent relationships between increasing PAMG‐1 levels and the risk of PTD within 1 and 2 weeks of CVF sampling.

## RESULTS

### Clinical characteristics

The characteristics of the singleton pregnancies included in this study are summarized in Table 1. The left side of the table represents the intragroup comparison for PTD within 1 week after CVF collection, while the right side of the table provides the intragroup comparison for PTD within 2 weeks after the collection. In both intragroup comparisons, PAMG1 and FFN exhibited significantly higher levels in the groups of PTD within 1 and 2 weeks. No significant differences were observed in the gestational age at which CVF samples were collected. Because this study focused on women with a short cervix, there were no significant differences in CL among the groups. Intragroup comparisons revealed significant differences in the rate of histological chorioamnionitis and neonatal/delivery characteristics, including gestational age at delivery, newborn birth weight, and Apgar scores, as expected. The characteristics of the twin pregnancies enrolled in this study are shown in Table S1, Supporting Information. In contrast to singleton pregnancies, PAMG‐1 and FFN levels in twin pregnancies were not significantly different.

**TABLE 1:** Maternal and neonatal/delivery characteristics of the study groups.

	Within 1‐week		*p*	Within 2‐week		*p*
	Delivery (*n* = 25)	No delivery (*n* = 107)		Delivery (*n* = 39)	No delivery (*n* = 93)	
Maternal characteristics						
Maternal age (years)	30.0 (29.0 to 34.0)	33.0 (30.0 to 36.0)	0.069	32.0 (29.5 to 36.0)	33.0 (30.0 to 35.0)	0.357
Primiparity	13 (52.0)	44 (41.1)	0.373	20 (48.7)	38 (40.9)	0.445
Infertility treatment	5 (20.0)	23 (21.5)	1.000	9 (23.1)	19 (20.4)	0.816
Pre‐pregnancy BMI	21.9 (19.1 to 23.6)	20.9 (18.2 to 22.9)	0.336	21.5 (19.1 to 23.5)	20.8 (18.1 to 22.9)	0.184
Pre‐pregnancy BMI ≥25	3 (12.0)	14 (13.1)	1.000	4 (10.3)	13 (14.0)	0.777
BMI at delivery	23.4 (21.0 to 25.1)	22.8 (21.3 to 26.4)	0.485	23.4 (21.1 to 25.1)	22.8 (21.2 to 26.5)	0.280
BMI at delivery ≥25	7 (28.0)	40 (37.4)	0.488	10 (25.6)	37 (39.8)	0.163
Smoking	2 (8.0)	1 (0.9)	0.092	2 (5.1)	1 (1.1)	0.208
History of preterm delivery	1 (4.0)	4 (3.7)	1.000	2 (5.1)	3 (3.2)	0.632
GA at CVF collection (weeks)	32.0 (28.6 to 33.3)	31.4 (29.7 to 33.3)	0.756	32.0 (28.4 to 33.3)	31.3 (29.7 to 33.3)	0.982
CL at CVF collection (mm)	14.0 (7.0 to 19.0)	13.0 (7.0 to 18.5)	0.785	12.0 (5.0 to 18.0)	13.0 (9.0 to 19.0)	0.156
FFN in CVF (ng/mL)	166 (86.0 to 242)	16.0 (16.0 to 94.0)	<0.001	121 (30.5 to 214)	16.0 (16.0 to 83.0)	<0.001
PAMG‐1 in CVF (pg/mL)	2193.1 (1616.0 to 2770.2)	829.1 (451.6 to 1406.2)	<0.001	2140.6 (1123.0 to 2901.3)	683.5 (451.6 to 1196.3)	<0.001
Histological chorioamnionitis	14 (56.0)	7 (12.2)^a^	<0.001	18 (47.4)^a^	3 (6.8)^a^	<0.001
Neonatal/delivery characteristics						
GA at delivery (weeks)	33.3 (29.6 to 34.0)	38.0 (36.4 to 39.9)	<0.001	33.3 (29.5 to 34.6)	38.6 (37.0 to 40.0)	<0.001
Cesarean section	10 (40.0)	28 (26.2)	0.220	15 (38.5)	23 (24.7)	0.141
Antenatal steroid treatment	15 (60.0)	47 (43.9)	0.183	27 (69.2)	35 (37.6)	<0.001
Birth weight (g)	1798 (1260 to 2048)	2742 (2506 to 3172)	<0.001	1858 (1336 to 2060)	2846 (2605 to 3172)	<0.001
Male	18 (72.0)	78 (77.2)^a^	0.605	25 (64.1)	71 (81.6)^a^	0.042
1‐min Apgar score <7	11 (44.0)	6 (5.6)	<0.001	16 (41.0)	1 (1.1)	<0.001
5‐min Apgar score <7	3 (12.0)	4 (3.7)	0.125	6 (15.4)	1 (1.1)	0.003
Umbilical artery pH <7.10	0 (0.0)	0 (0.0)	‐	0 (0.0)	0 (0.0)	‐
Umbilical artery BE	−2.6 (−3.5 to 0.4)	−2.2 (−3.2 to −0.7)	0.882	−2.7 (−4.0 to 0.3)	−2.1 (−3.2 to −0.7)	0.237

*Note*: Data are presented as medians (interquartile ranges) or *n* (%).

Abbreviations: BMI, body mass index; CL, cervical length; CVF, cervicovaginal fluid; FFN, fetal fibronectin; GA, gestational age; PAMG‐1, placental alpha microglobulin‐1.

^a^ Some data defects.

### Relationship of placental alpha macroglobulin‐1 to fetal fibronectin, and the impending preterm delivery

As shown in Table 2, the results of the multivariate logistic regression analyses indicated that positive PAMG‐1 was an independent associated factor for PTD within 1 to 2 weeks after CVF collection (for PTD within 1‐week, odds ratio [OR], 7.840; 95% CI, 2.020–30.50; *p* = 0.003: for PTD within 2‐week, OR, 7.340; 95%CI, 2.750–19.60; *p* < 0.001). Positive FFN was significantly associated with PTD within 1 week after CVF collection, but not with PTD within 2 weeks in this cohort.

**TABLE 2:** Associated factors with delivery within 1‐ or 2‐week by multivariate logistic regression analysis.

	Delivery within 1‐week			Delivery within 2‐week		
	OR	95% CI	*p*	OR	95% CI	*p*
Maternal characteristics						
Maternal age ≥35 years	0.530	0.145–1.930	0.335	0.897	0.323–2.490	0.835
Primiparity	1.110	0.372–3.320	0.850	1.150	0.462–2.840	0.769
Smoking	0.620	0.038–10.20	0.738	1.120	0.079–15.90	0.933
History of preterm labor	2.890	0.168–49.70	0.464	3.320	0.297–37.10	0.330
CL <15 mm	0.272	0.066–1.120	0.072	0.907	0.314–2.620	0.857
Positive FFN (≥50 ng/mL)	10.40	2.380–45.90	0.002	2.740	0.996–7.520	0.051
Positive PAMG‐1 (≥1000 pg/mL)	7.840	2.020–30.50	0.003	7.340	2.750–19.60	<0.001

Abbreviations: CI, confidence interval; CL, cervical length; FFN, fetal fibronectin; PAMG‐1, placental alpha microglobulin‐1; OR, odds ratio.

Table 3 shows the risk stratification for PTD within 1 week. The risk of PTD within 1 week increased in steps with higher PAMG‐1 levels (from 4.3% when PAMG‐1 was <1000 pg/mL to 50.0% when ≥3000 pg/mL) and higher FFN levels (from 6.5% when FFN was <50 ng/mL to 51.9% when ≥150 ng/mL). Notably, we found that there were no women with PTD within 1 week when both the PAMG‐1 and FFN test results were negative. Moreover, the highest rate of PTD within 1 week (66.7%) was observed when PAMG‐1 level was ≥2000 pg/mL and FFN level was ≥150 ng/mL. Table 4 shows the risk stratification for PTD within 2 weeks. The results were similar to those in Table 3, which showed an increased risk of PTD within 2 weeks with higher PAMG‐1 and FFN levels. Women with negative FFN were still identified as high risk with a positive PAMG‐1 test, and their rate of PTD within 2 weeks was 41.7%. Our findings also indicated that when PAMG‐1 was >3000 pg/mL, regardless of the FFN level, the rate of PTD within 2 weeks reaches 90%. Significant dose‐dependent trends were observed between increasing PAMG‐1 levels and the risk of PTD within both 1 week (*p* < 0.001) and 2 weeks (*p* < 0.001) according to the Cochran‐Armitage trend test, supporting a linear association between PAMG‐1 concentration and the PTD. Although the relatively small sample size may be a contributing factor, risk stratification through PAMG‐1 quantification, as observed in singleton pregnancies, was not found in women with twin pregnancies (Tables S2 and S3).

**TABLE 3:** Risk stratification of delivery within 1 week using quantitative PAMG‐1 and FFN combination.

Delivery within 1‐week			FFN			Total
			Negative	Positive		
			<50 ng/mL	50–149 ng/mL	≥150 ng/mL	
PAMG‐1	Negative	<1000 pg/mL	0.0 (0/53)	0.0 (0/8)	33.3 (3/9)	4.3 (3/70)
	Positive	1000–1999 pg/mL	17.6 (3/17)	14.3 (1/7)	50.0 (3/6)	23.3 (7/30)
		2000–2999 pg/mL	0.0 (0/3)	40.0 (4/10)	66.7 (6/9)	45.5 (10/22)
		≥3000 pg/mL	50.0 (2/4)	33.3 (1/3)	66.7 (2/3)	50.0 (5/10)
Total			6.5 (5/77)	21.4 (6/28)	51.9 (14/27)	18.9 (25/132)

Abbreviations: FFN, fetal fibronectin; PAMG‐1, placental alpha microglobulin‐1.

**TABLE 4:** Risk stratification of delivery within 2 weeks using quantitative PAMG‐1 and FFN combination.

Delivery within 2‐week			FFN			Total
			Negative	Positive		
			<50 ng/mL	50–149 ng/mL	≥150 ng/mL	
PAMG‐1	Negative	<1000 pg/mL	5.7 (3/53)	0.0 (0/8)	44.4 (4/9)	10.0 (7/70)
	Positive	1000–1999 pg/mL	29.4 (5/17)	14.3 (1/7)	66.7 (4/6)	33.3 (10/30)
		2000–2999 pg/mL	33.3 (1/3)	60.0 (6/10)	66.7 (6/9)	59.1 (13/22)
		≥3000 pg/mL	100.0 (4/4)	100.0 (3/3)	66.7 (2/3)	90.0 (9/10)
Total			16.9 (13/77)	35.7 (10/28)	59.3 (16/27)	29.5 (39/132)

Abbreviations: FFN, fetal fibronectin; PAMG‐1, placental alpha microglobulin‐1.

**FIGURE 1:**
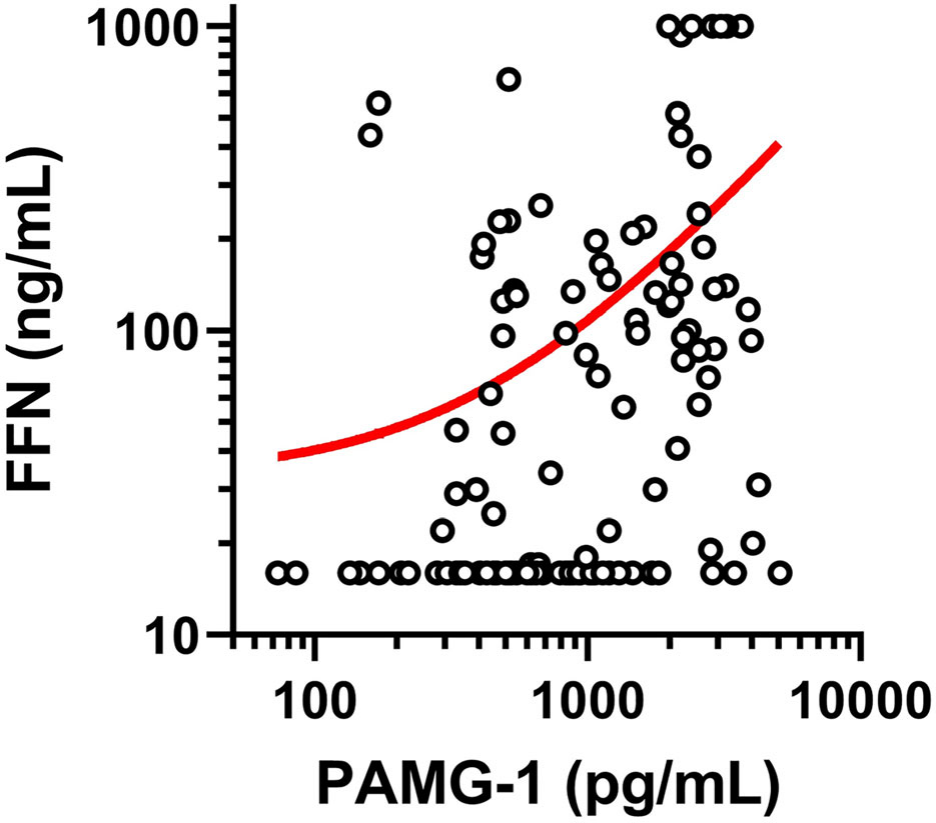
Dependence of placental alpha macroglobulin‐1 on fetal fibronectin. PAMG‐1 level has a slight association with fetal fibronectin levels (*p* < 0.001, *r* = 0.335).

Additionally, quantification of PAMG‐1 allowed us to evaluate its association with PAMG‐1 and FFN. As both PAMG‐1 and FFN serve as useful predictive markers for impending PTD, a significant association between the two markers was observed; however, the correlation coefficient was low (*p* < 0.001, *r* = 0.335) (Figure 1).

### Predictive ability of placental alpha macroglobulin‐1 for preterm delivery within 1‐ and 2‐week

As shown in Table 5, the predictive ability of the quantitative measurements of PAMG‐1 and FFN for impending PTD was evaluated. We also examined the potential of combining these markers in predicting impending PTD (Table 5). The results of the ROC analysis for PTD within 1 week are presented in the upper section of Table 5, while the results for PTD within 2 weeks are shown in the lower section of Table 5. All AUC values were >0.70, indicating favorable predictive performance. The combination of quantitative PAMG‐1 and FFN demonstrated the highest AUC values in both comparisons (0.845 for PTD within 1‐week and 0.804 for PTD within 2‐week). Although significant differences were not found in many factors, a statistical difference was observed between FFN alone and the combination of PAMG‐1 and FFN with respect to each specificity for the prediction of PTD within 2 weeks (66.7% and 83.9%, respectively). In addition, while the positive likelihood ratio (LR) remained modest across all models, the combination of PAMG‐1 and FFN showed a relatively higher positive LR for the 2‐week outcome, suggesting a trend toward improved discriminative power despite the lack of statistical significance.

**TABLE 5:** Receiver operating characteristic curves of three models for predicting delivery within 1 to 2 weeks.

	AUC (95% CI)	*p*	Sensitivity (95% CI)	*p*	Specificity (95% CI)	*p*	PPV (95% CI)	*p*	NPV (95% CI)	*p*	LR (+) (95% CI)	*p*	LR (−) (95% CI)	*p*
Delivery within 1‐week														
FFN	0.785 (0.687–0.883)	Ref.	60.0 (56.0–96.0)	Ref.	86.1 (57.0–90.7)	Ref.	50.0 (32.3–65.5)	Ref.	90.3 (89.1–98.6)	Ref.	4.32 (2.09–8.56)	Ref.	0.46 (0.10–52.8)	Ref.
PAMG‐1	0.797 (0.701–0.898)	0.857	72.0 (56.0–96.0)	0.602	79.4 (57.9–87.9)	0.200	45.0 (32.8–60.6)	0.631	92.4 (90.9–100)	0.800	3.50 (2.07–6.51)	0.120	0.35 (0.13–0.42)	0.354
PAMG‐1 + FFN	0.845 (0.767–0.924)	0.264	72.0 (68.0–100)	0.551	81.3 (59.8–88.8)	0.350	47.4 (34.0–61.2)	0.807	92.6 (91.6–100)	0.620	3.85 (2.19–6.58)	0.647	0.34 (0.15–0.36)	0.477
Delivery within 2‐week														
FFN	0.742 (0.651–0.833)	Ref.	79.5 (52.5–92.3)	Ref.	66.7 (55.9–92.5)	Ref.	50.0 (42.4–74.0)	Ref.	88.6 (80.7–95.6)	Ref.	2.39 (1.84–6.18)	Ref.	0.31 (0.12–0.57)	Ref.
PAMG‐1	0.788 (0.693–0.884)	0.458	82.1 (56.4–92.3)	1.000	68.8 (63.3–93.5)	0.875	52.5 (48.3–81.4)	0.857	90.1 (82.8–95.6)	0.792	2.63 (2.18–8.90)	0.681	0.26 (0.13–0.50)	0.729
PAMG‐1 + FFN	0.804 (0.717–0.892)	0.252	69.2 (59.0–92.3)	0.437	83.9 (63.5–94.1)	0.010	64.3 (49.2–80.9)	0.164	86.7 (83.4–96.3)	0.812	4.29 (2.25–10.3)	0.057	0.37 (0.11–0.48)	0.665

Abbreviations: AUC, area under the curve; CI, confidence interval; FFN, fetal fibronectin; LR, likelihood ratio; NPV, negative predictive value; PAMG‐1, placental alpha microglobulin‐1; PPV, positive predictive value; Ref., reference.

## DISCUSSION

This study is the first to evaluate the utility of quantitative assessment of PAMG‐1 in predicting impending PTD in asymptomatic women with a short cervix. By emphasizing its potential to refine risk categorization and improve predictive accuracy, particularly when combined with FFN, this study offers valuable insights into advancing the management of PTD.

Our findings confirmed that qualitative PAMG‐1 assessment, which is widely utilized with a standard threshold of 1000 pg/mL, is effective for predicting impending PTD. These findings support the clinical relevance of PAMG‐1 in predicting PTD even within Japanese clinical practice, where this test has not yet been routinely available. Furthermore, quantitative PAMG‐1 assessment provided additional precision, enabling a more detailed stratification of PTD risk among patients with positive results. The risk of impending PTD was found to increase progressively with higher PAMG‐1 levels. Quantitative assessment revealed a clear dose‐dependent relationship, with progressively higher risks observed at elevated PAMG‐1 concentrations for both PTD within 1‐ and 2‐week. This highlights the clinical significance of quantifying PAMG‐1, providing novel insights into its ability to stratify PTD risk, and a capability beyond the reach of qualitative tests alone. Quantitative assessment of PAMG‐1 enables a more nuanced risk stratification than qualitative tests alone. In clinical practice, reliable identification of women likely to deliver within a short‐time frame is critical for optimizing perinatal outcomes. Elevated quantitative levels of PAMG‐1, especially in combination with FFN, can guide timely administration of antenatal corticosteroids, magnesium sulfate, or maternal transfer to higher‐level care facilities. Conversely, lower concentrations may support decisions to avoid unnecessary hospitalization or interventions in women with low imminent risk. Thus, quantification may contribute not only to improved neonatal outcomes but also to more efficient and individualized maternal care strategies.

Distinct biological mechanisms underlie the contributions of PAMG‐1 and FFN to PTD: FFN levels rise due to disruption of the decidual‐chorionic interface, whereas PAMG‐1 levels increase through transudation across fetal membranes under uterine pressure.^11^, ^12^ These differences, supported by our findings, highlight the complementary nature of these biomarkers in predicting impending PTD. While PAMG‐1 retained predictive value even when FFN results were negative, positive FFN with negative PAMG‐1 results also indicated significant PTD risk. The combination of PAMG‐1 and FFN demonstrated the highest predictive accuracy, achieving AUC values of 0.845 for PTD within 1 week and 0.804 for PTD within 2 weeks, emphasizing the value of a dual‐marker approach. Our data show a significant but weak and negligible correlation between PAMG‐1 and FFN levels, further supporting the notion that these biomarkers reflect distinct aspects of PTD pathophysiology. This complementary relationship underscores the importance of PAMG‐1 quantification, which addresses mechanisms not captured by FFN alone. By refining PTD risk stratification, PAMG‐1 quantification represents a key contribution of this study, offering novel insights into biomarker‐based prediction strategies.

Previous studies have shown the benefits of quantitative FFN testing, demonstrating its superior accuracy compared to qualitative tests.^9^, ^13^, ^14^ Our study corroborates these findings, confirming that FFN levels ≥150 ng/mL are associated with a higher likelihood of PTD within 1 to 2 weeks. While prior research comparing PAMG‐1 and FFN primarily focused on qualitative assessments,^15^, ^16^ this study provides valuable insights into the utility of quantitative PAMG‐1 and its combined use with FFN, presenting a refined framework for PTD risk assessment.

Our study population was predominantly composed of third‐trimester cases, with 89.4% of CVF samples collected at between 28 and 34 weeks of gestation. An additional analysis excluding 14 singleton pregnancies sampled before 28 weeks indicates consistent results (Table S4), reinforcing the clinical homogeneity of our cohort. Therefore, our findings primarily reflect the utility of PAMG‐1 and FFN in the context of late PTD risk assessment, and should be interpreted accordingly.

Our findings show that, in twin pregnancies, both PAMG‐1 and FFN demonstrated limited predictive utility. A systematic review of 11 studies on asymptomatic women with multiple pregnancies concluded that FFN assessment provided only moderate to minimal prediction of PTD.^17^ These findings were further corroborated by a 2018 meta‐analysis by Dos Santos et al.,^18^ which included five studies on asymptomatic women with multiple pregnancies. Although a previous study reported a high negative predictive value of 96% for the qualitative PAMG‐1 test in twin pregnancies,^19^ our study did not identify effective risk stratification through PAMG‐1 quantification. This discrepancy may be attributed to the small sample size in our cohort, highlighting the necessity for further research to elucidate the role of these biomarkers in twin pregnancies.

Some limitations should be acknowledged. The study's retrospective design and relatively small sample size may limit its generalizability. It should be noted that the clinical implications of a short cervix may differ between singleton and twin pregnancies, and our findings should be interpreted accordingly. Additionally, while combining PAMG‐1 and FFN improves predictive accuracy, the cost‐effectiveness of implementing both tests routinely in clinical practice remains to be established. Although no clinical signs of membrane rupture were observed and nearly all participants had PAMG‐1 levels below 5 ng/mL, the presence of subclinical rupture of membranes cannot be definitively ruled out. Finally, as this study focused on women with short CLs (≤25 mm), further research is needed to assess its applicability to broader populations.

In conclusion, this study demonstrates for the first time the potential of quantitative PAMG‐1 assessment for predicting impending PTD. By allowing for more precise risk categorization and enhancing predictive accuracy when combined with FFN, PAMG‐1 quantification offers a promising advancement in PTD management. Future prospective, multicenter studies are essential to validate these findings and explore their broader clinical implications.

## AUTHOR CONTRIBUTIONS

**Yuki Nozaki:** Conceptualization; software; methodology; data curation; formal analysis; investigation; writing – original draft; visualization. **Kenji Imai:** Conceptualization; methodology; data curation; software; investigation; formal analysis; writing – original draft; visualization. **Rika Miki:** Formal analysis; project administration; investigation. **Sho Tano:** Formal analysis; project administration; investigation. **Kazuya Fuma:** Formal analysis; project administration; investigation. **Seiko Matsuo:** Formal analysis; project administration; investigation. **Takafumi Ushida:** Investigation; formal analysis; project administration. **Hiroaki Kajiyama:** Supervision; formal analysis; project administration; investigation. **Tomomi Kotani:** Supervision; formal analysis; project administration; investigation.

## CONFLICT OF INTEREST

Dr. Hiroaki Kajiyama is the Editor‐in‐Chief of the journal and the co‐author of this article. They were excluded from the peer‐review process and all editorial decisions related to the acceptance and publication of this article. Peer review was handled independently by the JOG Journal editorial office and Dr. Youichi Kobayashi as the Editor to minimize bias. The other authors have no competing interests to disclose.

## Supporting information


**Table S1.** Maternal and neonatal/delivery characteristics of the study groups (twin pregnancies).

**Table S2.** Risk stratification of delivery within 1 week using quantitative PAMG‐1 and FFN combination (twin pregnancies).

**Table S3.** Risk stratification of delivery within 2 weeks using quantitative PAMG‐1 and FFN combination (twin pregnancies).

**Table S4.** Maternal and neonatal/delivery characteristics of the singleton pregnant women sampled for CVF between 28 and 34 weeks of gestation.

## Data Availability

The clinical data were used solely for the purposes approved in this study protocol. Secondary use of the data by third parties is not permitted without additional review and approval by the ethics committee of Nagoya University. However, researchers who wish to access the data for legitimate academic purposes may contact the corresponding author at kenchan2@med.nagoya‐u.ac.jp. Each request will be individually reviewed and must undergo ethical evaluation.
