# Supplementary material for: Quantitative assessment of placental alpha macroglobulin‐1 for predicting impending preterm delivery in asymptomatic women with a short cervix
Source: J Obstet Gynaecol Res. 2025 Sep 5;51(9):e70071. doi: 10.1111/jog.70071 (PMC12413581; doi:10.1111/jog.70071)
Supplement: Supplementary file 1 — Table S1. Maternal and neonatal/delivery characteristics of the study groups (twin pregnancies). [file JOG-51-0-s001.docx]

| Supplemental Table 1. Maternal and neonatal/delivery characteristics of the study groups (twin pregnancies) | | | | | | | | |
| --- | --- | --- | --- | --- | --- | --- | --- | --- |
|  |  | Within 1-week | | *p* |  | Within 2-week | | *p* |
|  |  | Delivery (n = 8) | No Delivery (n = 72) |  |  | Delivery (n = 19) | No Delivery (n = 61) |  |
|  | Maternal age (years) | 34.5 (30.0 to 41.0) | 34.0 (31.0 to 38.3) | 0.897 |  | 32.0 (31.0 to 39.0) | 35.0 (32.0 to 39.0) | 0.532 |
|  | Primiparity | 1 (12.5) | 22 (30.6) | 0.427 |  | 4 (21.1) | 19 (31.1) | 0.563 |
|  | Infertility treatment | 6 (75.0) | 52 (72.2) | 1.000 |  | 15 (78.9) | 43 (70.5) | 0.566 |
|  | Pre-pregnancy BMI | 19.7 (18.1 to 22.1) | 21.1 (19.4 to 22.8) | 0.315 |  | 20.1 (18.2 to 22.0) | 21.2 (19.4 to 22.8) | 0.167 |
|  | BMI at birth | 21.6 (21.1 to 24.9) | 24.2 (22.8 to 27.3) | 0.055 |  | 21.9 (21.1 to 24.0) | 25.6 (23.1 to 27.6) | <0.001 |
|  | Smoking | 1 (12.5) | 12 (16.7) | 1.000 |  | 2 (10.5) | 11 (22.0) | 0.723 |
|  | History of preterm delivery | 0 (0.0) | 0 (0.0) | - |  | 0 (0.0) | 0 (0.0) | - |
|  | GA at CVF collection (weeks) | 32.4 (31.4 to 33.3) | 30.6 (27.6 to 32.4) | 0.060 |  | 31.7 (29.1 to 32.9) | 30.6 (27.7 to 32.4) | 0.398 |
|  | CL at CVF collection (mm) | 5.5 (5.0 to 10.0) | 13.0 (8.0 to 19.0) | 0.010 |  | 9.0 (5.0 to 11.5) | 13.0 (8.0 to 18.0) | 0.024 |
|  | FFN in CVF (ng/mL) | 166 (28.8 to 745) | 42.0 (16.0 to 121) | 0.104 |  | 33.0 (16.0 to 305.5) | 48.0 (16.0 to 107.0) | 0.488 |
|  | PAMG-1 in CVF (pg/mL) | 805.5 (550.2 to 1256.8) | 1177.3 (781.7 to 2357.3) | 0.202 |  | 1123.2 (782.3 to 2536.8) | 1123.2 (781.7 to 2338.4) | 0.888 |
|  | GA at delivery (weeks) | 32.9 (31.9 to 34.1) | 37.1 (33.7 to 37.6) | 0.004 |  | 32.3 (30.5 to 33.7) | 37.3 (37.0 to 37.6) | <0.001 |
|  | Histological chorioamnionitis | 0 (0.0)* | 0 (0.0)* | - |  | 0 (0.0)* | 0 (0.0)* | - |
| Data are presented as medians (interquartile ranges) or n (%). *some data defects. BMI: Body mass index, CVF: Cervicovaginal fluid, FFN: Fetal fibronectin, PAMG-1: Placental alpha microglobulin-1, GA: Gestational age, CL: Cervical length. | | | | | | | | |
