# Supplementary material for: Quantitative assessment of placental alpha macroglobulin‐1 for predicting impending preterm delivery in asymptomatic women with a short cervix
Source: J Obstet Gynaecol Res. 2025 Sep 5;51(9):e70071. doi: 10.1111/jog.70071 (PMC12413581; doi:10.1111/jog.70071)
Supplement: Supplementary file 2 — Table S2. Risk stratification of delivery within 1 week using quantitative PAMG‐1 and FFN combination (twin pregnancies). [file JOG-51-0-s003.docx]

| Supplemental Table 2. Risk stratification of delivery within 1 week using quantitative PAMG-1 and FFN combination (twin pregnancies) | | | | | | |
| --- | --- | --- | --- | --- | --- | --- |
| Delivery within 1-week | | | FFN | | | Total |
|  |  |  | Negative | Positive | |  |
|  |  |  | < 50 ng/mL | 50 -149 ng/mL | ≥ 150 ng/mL |  |
| PAMG-1 | Negative | < 1000 pg/mL | 11.8 | 12.5 | 30.0 | 17.1 |
|  |  |  | (2/17) | (1/8) | (3/10) | (6/35) |
|  | Positive | 1000 - 1999 pg/mL | 0.0 | 0.0 | 0.0 | 0.0 |
|  |  |  | (0/11) | (0/5) | (0/3) | (0/19) |
|  |  | 2000 - 2999 pg/mL | 0.0 | 0.0 | 25.0 | 8.3 |
|  |  |  | (0/6) | (0/2) | (1/4) | (1/12) |
|  |  | ≥ 3000 pg/mL | 14.3 | 0.0 | 0.0 | 7.1 |
|  |  |  | (1/7) | (0/3) | (0/4) | (1/14) |
| Total | | | 7.3 | 5.5 | 19.0 | 10.0 |
|  |  |  | (3/41) | (1/18) | (4/21) | (8/80) |
| PAMG-1: Placental alpha microglobulin-1, FFN: Fetal fibronectin. | | | | | | |
