# Supplementary material for: Quantitative assessment of placental alpha macroglobulin‐1 for predicting impending preterm delivery in asymptomatic women with a short cervix
Source: J Obstet Gynaecol Res. 2025 Sep 5;51(9):e70071. doi: 10.1111/jog.70071 (PMC12413581; doi:10.1111/jog.70071)
Supplement: Supplementary file 3 — Table S3. Risk stratification of delivery within 2 weeks using quantitative PAMG‐1 and FFN combination (twin pregnancies). [file JOG-51-0-s002.docx]

| Supplemental Table 3. Risk stratification of delivery within 2 weeks using quantitative PAMG-1 and FFN combination (twin pregnancies) | | | | | | |
| --- | --- | --- | --- | --- | --- | --- |
| Delivery within 2-week | | | FFN | | | Total |
|  |  |  | Negative | Positive | |  |
|  |  |  | < 50 ng/mL | 50 -149 ng/mL | ≥ 150 ng/mL |  |
| PAMG-1 | Negative | < 1000 pg/mL | 22.2 | 12.5 | 40.0 | 25.0 |
|  |  |  | (4/18) | (1/8) | (4/10) | (9/36) |
|  | Positive | 1000 - 1999 pg/mL | 30.0 | 20.0 | 0.0 | 22.2 |
|  |  |  | (3/10) | (1/5) | (0/3) | (4/18) |
|  |  | 2000 - 2999 pg/mL | 0.0 | 0.0 | 50.0 | 16.7 |
|  |  |  | (0/6) | (0/2) | (2/4) | (2/12) |
|  |  | ≥ 3000 pg/mL | 42.9 | 0.0 | 25.0 | 28.6 |
|  |  |  | (3/7) | (0/3) | (1/4) | (4/14) |
| Total | | | 24.4 | 11.1 | 33.3 | 23.8 |
|  |  |  | (10/41) | (2/18) | (7/21) | (19/80) |
| PAMG-1: Placental alpha microglobulin-1, FFN: Fetal fibronectin. | | | | | | |
