# Supplementary material for: Quantitative assessment of placental alpha macroglobulin‐1 for predicting impending preterm delivery in asymptomatic women with a short cervix
Source: J Obstet Gynaecol Res. 2025 Sep 5;51(9):e70071. doi: 10.1111/jog.70071 (PMC12413581; doi:10.1111/jog.70071)
Supplement: Supplementary file 4 — Table S4. Maternal and neonatal/delivery characteristics of the singleton pregnant women sampled for CVF between 28 and 34 weeks of gestation. [file JOG-51-0-s004.docx]

| Supplemental Table4. Maternal and neonatal/delivery characteristics of the singleton pregnant women sampled for CVF between 28 and 34 weeks of gestation | | | | | | | | |  |
| --- | --- | --- | --- | --- | --- | --- | --- | --- | --- |
|  |  | Within 1-week | | *p* |  | Within 2-week | | *p* |  |
|  |  | Delivery (n = 20) | No Delivery (n = 98) |  |  | Delivery (n = 31) | No Delivery (n = 87) |  |  |
| *Maternal characteristics* | |  |  |  |  |  |  |  |  |
|  | Maternal age (years) | 30.0 (29.8 to 33.5) | 33.0 (30.0 to 36.0) | 0.078 |  | 30.0 (29.5 to 36.0) | 33.0 (30.0 to 35.0) | 0.230 |  |
|  | Primiparity | 10 (50.0) | 41 (41.8) | 0.622 |  | 14 (45.2) | 37 (42.5) | 0.835 |  |
|  | Infertility treatment | 5 (25.0) | 20 (20.4) | 0.764 |  | 7 (22.6) | 18 (20.7) | 0.803 |  |
|  | Pre-pregnancy BMI | 22.0 (18.6 to 23.5) | 20.9 (18.2 to 22.9) | 0.620 |  | 21.9 (18.9 to 23.1) | 20.9 (18.1 to 23.0) | 0.487 |  |
|  | Pre-pregnancy BMI ≥25 | 3 (15.0) | 14 (14.3) | 1.000 |  | 4 (12.9) | 13 (14.9) | 1.000 |  |
|  | BMI at delivery | 23.4 (20.9 to 25.3) | 22.8 (21.3 to 26.5) | 0.337 |  | 23.4 (21.2 to 25.3) | 22.8 (21.3 to 26.7) | 0.199 |  |
|  | BMI at delivery ≥25 | 6 (30.0) | 40 (40.8) | 0.455 |  | 9 (29.0) | 37 (42.5) | 0.206 |  |
|  | Smoking | 2 (10.0) | 1 (1.0) | 0.074 |  | 2 (6.5) | 1 (1.1) | 0.168 |  |
|  | History of preterm delivery | 1 (5.0) | 3 (3.1) | 0.529 |  | 2 (6.5) | 2 (2.3) | 0.282 |  |
|  | GA at CVF collection (weeks) | 32.7 (30.2 to 33.4) | 31.9 (30.3 to 33.3) | 0.451 |  | 32.9 (31.4 to 33.5) | 31.7 (30.3 to 33.3) | 0.128 |  |
|  | CL at CVF collection (mm) | 15.5 (7.8 to 20.3) | 13.0 (7.0 to 18.0) | 0.269 |  | 14.0 (6.0 to 19.0) | 13.0 (8.0 to 18.5) | 0.849 |  |
|  | FFN in CVF (ng/mL) | 166 (72.0 to 294) | 16.0 (16.0 to 93.0) | <0.001 |  | 117 (30.0 to 226) | 16.0 (16.0 to 89.0) | <0.001 |  |
|  | PAMG-1 in CVF (pg/mL) | 2193.1 (1874.3 to 2717.7) | 829.5 (454.6 to 1432.4) | <0.001 |  | 2140.6 (1159.6 to 2770.2) | 695.7 (451.6 to 1251.0) | <0.001 |  |
|  | Histological chorioamnionitis | 9 (45.0) | 4 (7.7) * | 0.001 |  | 11 (36.7) * | 2 (4.8) * | 0.001 |  |
| *Neonatal/delivery characteristics* | |  |  |  |  |  |  |  |  |
|  | GA at delivery (weeks) | 33.5 (30.4 to 34.3) | 38.0 (36.7 to 39.9) | <0.001 |  | 33.7 (32.9 to 34.6) | 38.6 (37.0 to 40.0) | <0.001 |  |
|  | Cesarean section | 7 (35.0) | 25 (25.5) | 0.414 |  | 10 (32.3) | 22 (25.3) | 0.485 |  |
|  | Antenatal steroid treatment | 10 (50.0) | 41 (41.8) | 0.622 |  | 19 (61.3) | 32 (36.8) | 0.021 |  |
|  | Birth weight (g) | 1881 (1624 to 2060) | 2802 (2506 to 3172) | <0.001 |  | 2140 (1160 to 2770) | 2846 (2594 to 3172) | <0.001 |  |
|  | Male | 13 (65.0) | 73 (79.3) * | 0.240 |  | 19 (61.3) | 67 (82.7) * | 0.024 |  |
|  | 1-min Apgar score | 8.0 (6.0 to 8.0) | 8.0 (8.0 to 9.0) | <0.001 |  | 8.0 (6.0 to 8.0) | 8.0 (8.0 to 9.0) | <0.001 |  |
|  | 1-min Apgar score < 7 | 7 (35.0) | 4 (4.1) | <0.001 |  | 11 (35.5) | 0 (0.0) | <0.001 |  |
|  | 5-min Apgar score | 8.0 (8.0 to 9.0) | 9.0 (9.0 to 9.0) | <0.001 |  | 9.0 (8.0 to 9.0) | 9.0 (9.0 to 9.0) | <0.001 |  |
|  | 5-min Apgar score < 7 | 1 (5.0) | 2 (2.0) | 0.430 |  | 3 (9.7) | 0 (0.0) | 0.017 |  |
|  | Umbilical artery pH | 7.38 (7.33 to 7.39) | 7.33 (7.28 to 7.39) | 0.091 |  | 7.35 (7.32 to 7.39) | 7.33 (7.28 to 7.38) | 0.234 |  |
|  | Umbilical artery pH < 7.10 | 0 (0.0) | 0 (0.0) | - |  | 0 (0.0) | 0 (0.0) | - |  |
|  | Umbilical artery BE | -3.1 (-3.6 to -1.8) | -2.2 (-3.2 to -0.7) | 0.180 |  | -3.3 (-4.9 to -1.4) | -2.1 (-3.2 to -0.7) | 0.020 |  |
| Data are presented as medians (interquartile ranges) or n (%). *Some data defects. BMI: Body mass index, CVF: Cervicovaginal fluid, FFN: Fetal fibronectin, PAMG-1: Placental alpha microglobulin-1, GA: Gestational age, CL: Cervical length. | | | | | | | | |  |
